# Circulating extracellular vesicles from severe COVID-19 patients induce lung inflammation

**DOI:** 10.1128/msphere.00764-24

**Published:** 2024-10-30

**Authors:** Huifeng Qian, Ruoxi Zang, Ruoyang Zhang, Guoping Zheng, Guanguan Qiu, Jianbiao Meng, Jiangmei Wang, Jie Xia, Ruoqiong Huang, Zhenkai Le, Qiang Shu, Jianguo Xu

**Affiliations:** 1 Shaoxing Second Hospital, Shaoxing, Zhejiang, China; 2 National Clinical Research Center for Child Health, The Children’s Hospital of Zhejiang University School of Medicine, Hangzhou, Zhejiang, China; 3 Tongde Hospital of Zhejiang, Hangzhou, Zhejiang, China; Johns Hopkins University Bloomberg School of Public Health, Baltimore, Maryland, USA

**Keywords:** COVID-19, extracellular vesicles, lung inflammation, macrophage polarization

## Abstract

Circulating extracellular vesicles (EVs) have been associated with the development of COVID-19 due to their roles in viral infection, inflammatory response, and thrombosis. However, the direct induction of lung inflammation by circulating EVs from severe COVID-19 patients remains unknown. EVs were extracted from the plasma of severe COVID-19 patients admitted to intensive care and healthy controls. To study the effect of COVID-19 EVs on lung inflammation, mice were intratracheally instilled with EVs. To examine the proinflammatory effects of EVs *in vitro*, bone marrow-derived macrophages were treated with EVs. COVID-19 but not control EVs triggered lung inflammation, as assessed by total protein level, total cell count, neutrophil count, and levels of proinflammatory cytokines in the bronchoalveolar lavage. COVID-19 EVs also promoted M1 polarization of alveolar macrophages *in vivo*. Treatment of bone marrow-derived macrophages with COVID-19 EVs enhanced the M1 phenotype and augmented the production of IL-1β, IL-6, and TNF-α. In summary, circulating EVs from severe COVID-19 patients induce lung inflammation in mice. EVs could become a potential therapeutic target for alleviating lung injury in COVID-19.

**IMPORTANCE:**

Extracellular vesicles (EVs) have been reported to facilitate cytokine storm, coagulation, vascular dysfunction, and the spread of the virus in COVID-19. The direct role of circulating EVs from severe COVID-19 patients in lung injury remains unrecognized. Our study demonstrated that plasma EVs obtained from severe COVID-19 patients induced lung inflammation and polarization of alveolar macrophages *in vivo*. *In vitro* experiments also revealed the proinflammatory effects of COVID-19 EVs. The present study sheds fresh insight into the mechanisms of COVID-19-induced lung injury, highlighting EVs as a potential therapeutic target in combating the disease.

## INTRODUCTION

The COVID-19 pandemic led to a global health crisis from 2020 to 2023. Acute respiratory distress syndrome (ARDS) is the most severe complication of COVID-19, affecting up to 90% of the non-survivors (1). COVID-19-associated ARDS primarily results from the interaction between the severe acute respiratory syndrome coronavirus 2 (SARS-CoV-2) and the host’s immune response, culminating in acute lung injury (2). The genome of SARS-CoV-2 encodes four structural proteins including spike (S), envelope, membrane, and nucleocapsid (N). S protein binds to angiotensin-converting enzyme 2 (ACE2) on host cells via receptor-binding domain (3). ACE2 exhibits high expression in alveolar type 2 (AT2) cells and low expression in AT1 cells, basal cells, club cells, and alveolar macrophages (4–6). After ACE2 binding, transmembrane serine protease 2 cleaves the S protein, resulting in the integration of the viral and cell membranes and subsequent delivery of the viral RNA genome to the host cells (7). The direct impact of SARS-CoV-2 viral replication causes damage and injury to the alveoli. The viral replication also stimulates the production of proinflammatory cytokines and activation of alveolar macrophages, resulting in the recruitment of neutrophils and cytokine storm (8). The cytokine storm induces epithelial damage, endothelial injury, and elevated coagulation and vascular permeability, culminating in acute lung injury (9).

Extracellular vesicles (EVs) are lipid bilayer membrane vesicles released by almost all cell types. EVs were originally considered as a mechanism of waste removal for cells and are now understood as an essential player in both physiological and pathological processes. They provide a mechanism for intercellular communication by promoting the exchange of protein, mRNA, DNA, and miRNA (10). EVs have been proposed as potential biomarkers and therapeutic options for COVID-19 (11, 12). Balbi et al. (13) reported that circulating EVs from COVID-19 patients had enhanced procoagulant activity, resulting in increased disease severity. Rosell et al. (14) found that tissue factor (TF) activity in EVs of COVID-19 patients was higher than that of healthy controls and correlated with severity and mortality. Zipperle et al. (15) showed that endothelial EVs as well as miRNAs (MiR-223-3p, miR-191-5p, and miR-126-3p) in EVs displayed higher expression in the ICU group of COVID-19 patients. Suades et al. (16) discovered that CD66b^+^/CD68^+^ EVs could differentiate severe from non‐severe COVID‐19. Xia et al. (17) documented that COVID-19 EVs contained substantial quantities of live virus particles. In addition, EVs were internalized by recipient cells directly, indicating the role of EVs in cell-to-cell viral transmission. Circulating EVs expressing ACE2 were elevated in COVID-19 patients and correlated with disease severity. These EVs not only neutralized SARS-CoV-2 infection but also alleviated virus-induced lung injury in transgenic mice expressing human ACE2 (18). Proteomic analysis showed that the protein content of EVs during COVID-19 convalescence was associated with the severity of the original disease. EVs from mild convalescent COVID-19 subjects presented a suppressive effect on T cell effector function (19). Circulating EVs derived from COVID-19 and long-COVID patients were found to contain structural proteins S and N, while abnormal levels of several mitochondrial proteins in EVs during acute infection predicted long-COVID (20).

The pathophysiological mechanisms underlying SARS-CoV-2-induced lung injury are quite complex and require further exploration. The causative relationship between circulating EVs from severe COVID-19 patients and lung inflammation remains unknown. In the present study, we hypothesized that EVs from severe COVID-19 patients may directly induce lung inflammation and M1 polarization of alveolar macrophages.

## MATERIALS AND METHODS

### Study subjects

Our study enrolled 13 COVID-19 patients at the ICU of Shaoxing Second Hospital, Zhejiang, China between 20 December 2022 and 20 January 2023. During that time, China had a massive Omicron outbreak after easing control measures. COVID-19 was diagnosed by polymerase chain reaction using samples from nasopharyngeal swabs. Exclusion criteria included (i) age less than 18 or pregnancy; (ii) patients on immunosuppressive therapy or chemotherapy; (iii) HBV, HCV, or HIV infection; (iv) autoimmune or chronic inflammatory diseases. Peripheral blood samples of 13 severe COVID-19 patients were obtained within 48 h following admission to ICU. Samples from 13 age- and sex-matched healthy controls were included in the study. Informed consent was collected from all participants or their legal representatives. Demographic characteristics and clinical data were extracted from the medical record for study purposes.

### Isolation of EVs from plasma

Samples of venous blood were gathered into tubes containing EDTA and centrifuged within 4 h of sample collection at 800 *g* for 10 min. Plasma samples were carefully collected and preserved at −80°C. On the day of extraction for EVs, samples were thawed and diluted with EV-depleted phosphate-buffered saline (PBS). The mixture was centrifuged at 10,000 *g* for 30 min using a SW32 Ti swinging-bucket rotor (Beckman Coulter, Brea, CA, USA). Afterward, the upper fraction was transferred to a new SW32 Ti centrifuge tube and centrifuged at 120,000 *g* for 120 min. The pellet was rinsed with EV-depleted PBS, moved to a SW 41 Ti tube, and subjected to a second 120,000 *g* for 120 min. The resulting pellet was dissolved with 50 µL of PBS. The EV yield was assessed by quantifying the protein concentration utilizing the Pierce BCA Protein Detection Kit (Thermo Fisher Scientific, Waltham, MA, USA).

### Transmission electron microscopy

Transmission electron microscopy (TEM) was utilized to acquire the shape and morphology of EVs, with support from the EM core facility at Zhejiang University. After dilution with PBS, EV samples were applied onto 200 mesh copper grids with Formvar support film (Electron Microscopy Sciences, Hatfield, PA, USA) for 2 min. Uranyl acetate solution (2%) was added to the grids for negative staining of EVs. Excess staining was drained by blotting with filter paper after 1 min. After drying, the specimens were ready for observation under a 120 kV TEM microscope (FEI Tecnai G2 Spirit, Hillsboro, OR, USA).

### Nanoparticle tracking analysis

The concentration and diameter of EVs were examined utilizing a ZetaView PMX 110 instrument (Particle Metrix, Meerbush, Germany) following instructions from the manufacturer. EV samples were diluted with particle-free PBS to achieve a concentration range of 10^6^–10^9^ particles/mL. A small volume of specimen, free of air bubbles, was loaded into the sample chamber. Detection was performed at the following camera settings: autofocus, sensitivity at 70, and shutter at 100. The recorded videos were analyzed with ZetaView software (version 8.2.30.1) with the following settings: maximum size of 1,000 nm, minimum size of 10 nm, and minimum brightness of 25.

### Mouse model of EV-induced lung inflammation

C57BL/6 mice in the study were procured from the Shanghai Laboratory Animal Center (Shanghai, China) and given 1 week to acclimate to the new environment before starting the experiment. Mice were maintained under 12-h day/night cycle and provided with food and water *ad libitum*. Mice were then randomly assigned to treatment groups and anesthetized with sodium phenobarbital (50 mg/kg of body weight, intraperitoneal). Intratracheal instillation was performed with either EVs (100 µg) or PBS in a volume of 50 µL. Mice were sacrificed after 24 h, and lung samples along with bronchoalveolar lavage (BAL) were harvested for further analysis.

### Bone marrow-derived macrophages

Femurs from 6- to 8-week-old C57BL/6 mice were flushed with PBS using 25-gauge needles. Bone marrow cells were harvested by centrifugation for 5 min at 300 *g*. Cells were cultured in Dulbecco's modified Eagle medium (DMEM) supplemented with 10% FBS and 20 ng/mL GM-CSF (differentiation medium). In the following morning, non-adherent cells were transferred to 6-well plates and supplied with fresh differentiation medium. On day 7, mature bone marrow-derived macrophages (BMDMs) were harvested from the culture and transferred to 12-well plates with 5 × 10^5^ cells/well overnight. BMDMs were primed with EVs (100 µg/mL) from control or COVID-19 patients for 24 h. Subsequently, cells were harvested and analyzed by flow cytometry. The culture supernatant was examined for levels of IL-1β, IL-6, and TNF-α via ELISA (MultiSciences, Hangzhou, China) according to the manufacturer’s instructions.

### Examination of lung injury using BAL

BAL fluid was collected from mice 24 h post-treatment with EVs by flushing the airway three times with 0.6 mL of PBS. The fluid was subjected to centrifugation at 500 *g* for 5 min. The resulting supernatant was assayed for protein concentration via the Pierce BCA Protein Assay Kit (Thermo Fisher). The pellets were examined for total white blood cells utilizing a hemocytometer. Neutrophils were quantified by flow cytometry after staining with an anti-Ly6G antibody (Thermo Fisher). Additionally, levels of IL-1β, IL-6, and TNF-α in the BAL supernatant were assayed using ELISA.

### Flow cytometry

For the characterization of macrophage polarization in BAL and BMDMs, cells were blocked by incubating with 0.5% BSA in PBS (staining buffer). Afterward, cells were incubated with anti-mouse PE-F4/80 and BV650-CD86 (BD Biosciences, Franklin Lakes, NJ, USA) or isotype controls diluted in staining buffer for 30 min. Following staining, unbound antibodies were washed off with staining buffer. To perform intracellular staining of iNOS and CD206, cells were incubated for 30 min with Fixation/Perm working buffer (Thermo Fisher). Then, cells were stained with anti-mouse BV785 CD206 and PE-cy7 iNOS (BioLegend, San Diego, CA, USA) or their isotype controls diluted in staining buffer for 30 min on ice. Cells were washed again to remove unbound antibodies and fixed with 4% paraformaldehyde. Stained cells were analyzed on a BD LSRFortessa for data acquisition. Analysis of flow cytometry data was conducted using FlowJo V10 software.

### Histopathology

Following euthanasia, an 18-gauge cannula was carefully inserted into the trachea and secured using a ligature. The lungs were gently inflated via the cannula with 4% buffered paraformaldehyde. Immediately, the lungs were removed and placed in a jar containing the same paraformaldehyde solution to fix for 24 h. The fixed lung tissue was processed with a gradient series of ethanol, embedded in paraffin, and sectioned into 5 µm in thickness. Subsequently, tissue sections were subjected to paraffin removal with xylene and rehydration with an ethanol series (100% for 5 min; 95% for 5 min; and 70% for 5 min). To visualize lung structure, hematoxylin and eosin (H and E) staining was conducted using standard protocol. Images of lung sections were taken by a camera mounted to an Olympus VS120 microscope (Shinjuku, Tokyo, Japan).

### Western blot

Plasma EVs were treated with lysis buffer at 4°C for 30 min. EVs and total plasma samples (10 µg) were separated by electrophoresis on a Novex Tris-Glycine gel (Thermo Fisher) and transferred to membranes. The membranes were blocked in milk buffer (5% milk in Tris-buffered saline with Tween-20), then probed with primary antibody for CD9 (Cat #ab263019, Abcam, Waltham, MA, USA), TSG101 (Cat #67381-1-Ig, Proteintech, Wuhan, China), ApoE (Cat# 66830-1-Ig, Proteintech), and Calnexin (Cat #66903-1-Ig, Proteintech) overnight at 4°C. After washing, membranes were exposed to secondary antibodies for 60 min. Signals were detected using chemiluminescence.

### Statistical analysis

GraphPad Prism 8.0.2 software was applied for statistical analysis. Data were tested for their normality using in-built software and are shown as mean and standard deviation (SD) for continuous variables. Differences between two continuous variables was assessed with the Student’s two-tailed *t* test. Difference among three or more continuous variables was tested with a one-way analysis of variance followed by Tukey’s *post hoc* analysis. A *P* value of less than 0.05 was regarded as statistically significant.

## RESULTS

### Study patient characteristics

China had a rapid and extensive Omicron outbreak between December 2022 and January 2023 as the result of easing of control measures. Approximately 97% of the population was infected during this period (21). Thirteen ICU patients diagnosed with severe COVID-19 and 13 age- and sex-matched healthy controls were recruited for the study. Table 1 shows the demographic data, clinical characteristics at ICU admission, and outcomes of the study subjects. Healthy controls (72.6 ± 9.7 years) were slightly younger than the severe COVID-19 patients (74.8 ± 10.4 years). Nevertheless, the age difference did not yield statistical significance (*P* > 0.05). Severe COVID-19 patients displayed high levels of biomarkers for acute phase inflammation (C-reactive protein) and hypercoagulability (D-dimer). The patients also presented with high scores on the WHO COVID-19 Clinical Progression Scale and sequential organ failure assessment (SOFA) and had a high rate of mortality (76.9%).

**TABLE 1:** Demographic and clinical characteristics of severe COVID-19 patients^*a*^

Variable	COVID-19 (*n* = 13)	Healthy controls (*n* = 13)	*P*-value
Age (years)	74.8 ± 10.4	72.6 ± 9.7	>0.05
Male/female	7/6	7/6	
WHO COVID-19 Clinical Progression Scale	6.6 ± 0.96		
SOFA score	10.5 ± 2.7		
C-reactive protein (mg/L)	64.3 ± 35.5	–^*b*^	
D-dimer (mg/L)	5.5 ± 8.6	–	
Bilateral pneumonia (%)	13 (100%)	–	
ICU length of stay (days)	15.3 ± 12.4	–	
Death (*n*, %)	10 (76.9)	–	

^*a*^ Data are presented as patient number (%) or mean ± SD.

^*b*‘^ –’ indicates not available.

### Characteristics of plasma EVs from severe COVID-19 patients

**Fig 1:**
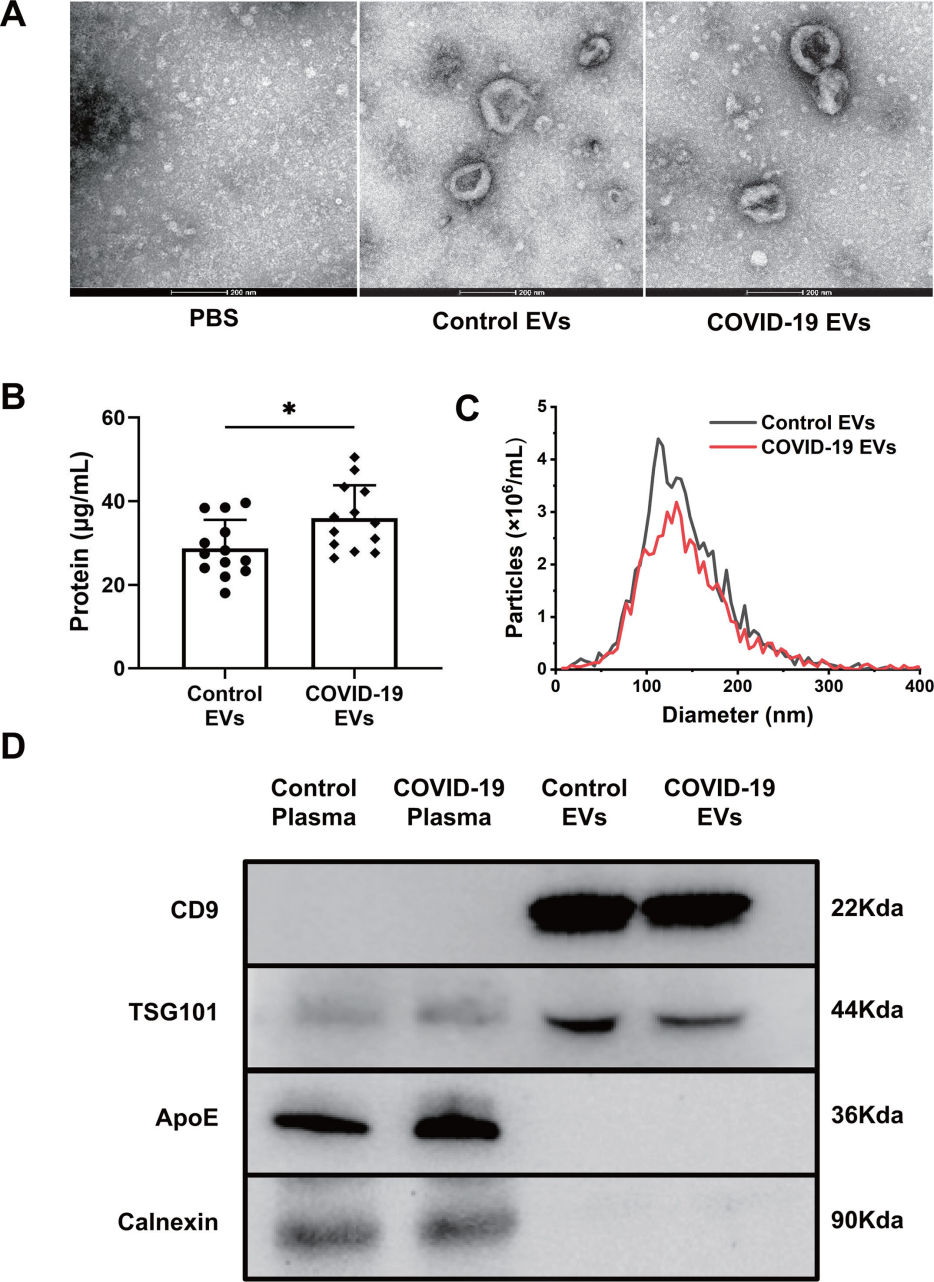
Characteristics of circulating EVs from COVID-19 patients. Differential centrifugation was used to separate plasma EVs collected from severe COVID-19 patients and healthy controls. (**A**) Images of circulating EVs were obtained by TEM. PBS was used as a negative control. (**B**) Concentration of total protein in EVs was determined by BCA protein detection kit and expressed as micrograms of protein per milliliter of plasma. Data are presented as mean  ±  SD. *n* = 13, *P < 0.05. (**C**) Size distribution graph of EVs was measured by NTA. (**D**) Total plasma and EVs were examined for the expression of EV markers (CD9 and TSG101) and non-EV markers (ApoE and calnexin) by Western blot.

Circulating EVs from severe COVID-19 patients and healthy controls were isolated by ultracentrifugation and imaged via TEM (Fig. 1A). COVID-19 EVs exhibited a higher protein concentration in comparison to EVs from healthy controls (*P* < 0.05, Fig. 1B). The size and distribution of EVs were determined by nanoparticle tracking analysis (NTA). COVID-19 EVs had a slight increase in peak size (132 nm) and a size range of 50–300 nm (Fig. 1C). Compared to total plasma, both types of EVs exhibited characteristic markers CD9 and TSG101, while the contamination markers such as ApoE and Calnexin were absent (Fig. 1D) (raw images in Fig. S1).

### Effects of COVID-19 EVs on lung inflammation in mice

**Fig 2:**
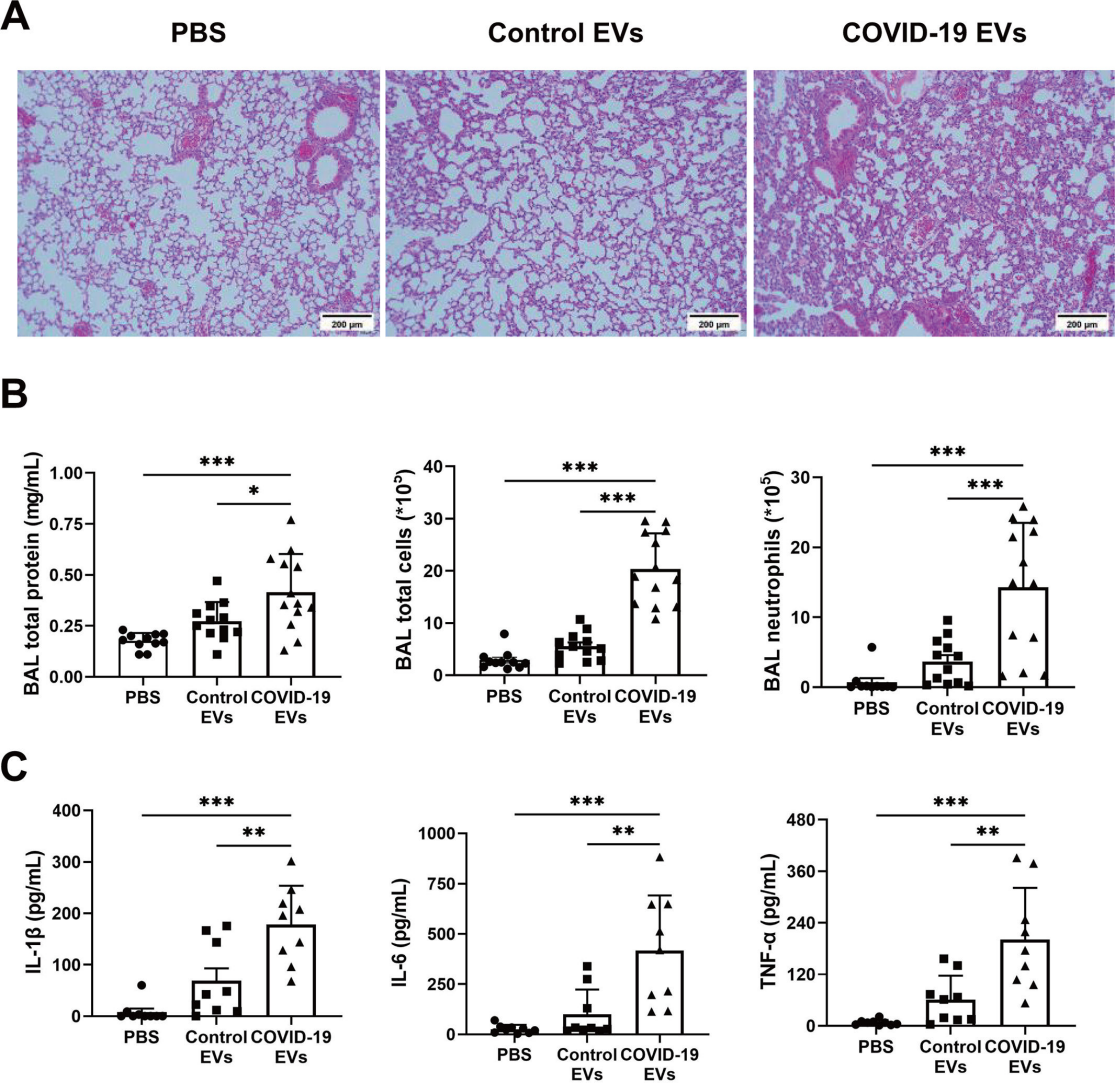
Circulating EVs from severe COVID-19 patients induce lung inflammation. C57BL/6 mice received an intratracheal injection of PBS (50 µL), control EVs (100 µg/50 µL), or COVID-19 EVs (100 µg/50 µL). (**A**) Lung sections were stained with H and E. (**B**) Lung injury was assessed by measuring total protein concentrations, total cell counts, and neutrophil counts in the BAL. *n* = 11–13. (**C**) Levels of IL-1β, IL-6, and TNF-α in BAL were examined via ELISA. *n* = 9. Each point represents an individual animal. Data are presented as mean ± SD. *P < 0.05, **P < 0.01, and ***P < 0.001.

Proteomic analysis of COVID-19 EVs conducted by Aharon et al. (22) identified several molecules in inflammation, coagulation, and complement pathways, suggesting that EVs could play a significant role in tissue damage. To study whether COVID-19 EVs induce lung injury *in vivo*, mice were treated with a single dose of PBS, control EVs, or COVID-19 EVs (100 µg/mouse in 50 µL) intratracheally. At 24 h, lung tissue and BAL were collected for analysis. Histological examination of lung sections showed that mice that received COVID-19 EVs had increased cellularity and septal thickness compared to mice that received control EVs or PBS (Fig. 2A). Mice exposed to COVID-19 EVs had higher protein concentrations, total cells, and neutrophils in the BAL than mice that received control EVs or PBS, indicating elevated vascular permeability and neutrophil infiltration (Fig. 2B). Lung inflammation was also studied by measuring cytokine levels in the BAL. The COVID-19 EVs group had increased levels of IL-1β, IL-6, and TNF-α compared to the other two groups (Fig. 2C). There was no difference between the control EVs group and the PBS group in all experiments (Fig. 2A through C).

### Effects of COVID-19 EVs on M1 polarization of alveolar macrophages *in vivo*

**Fig 3:**
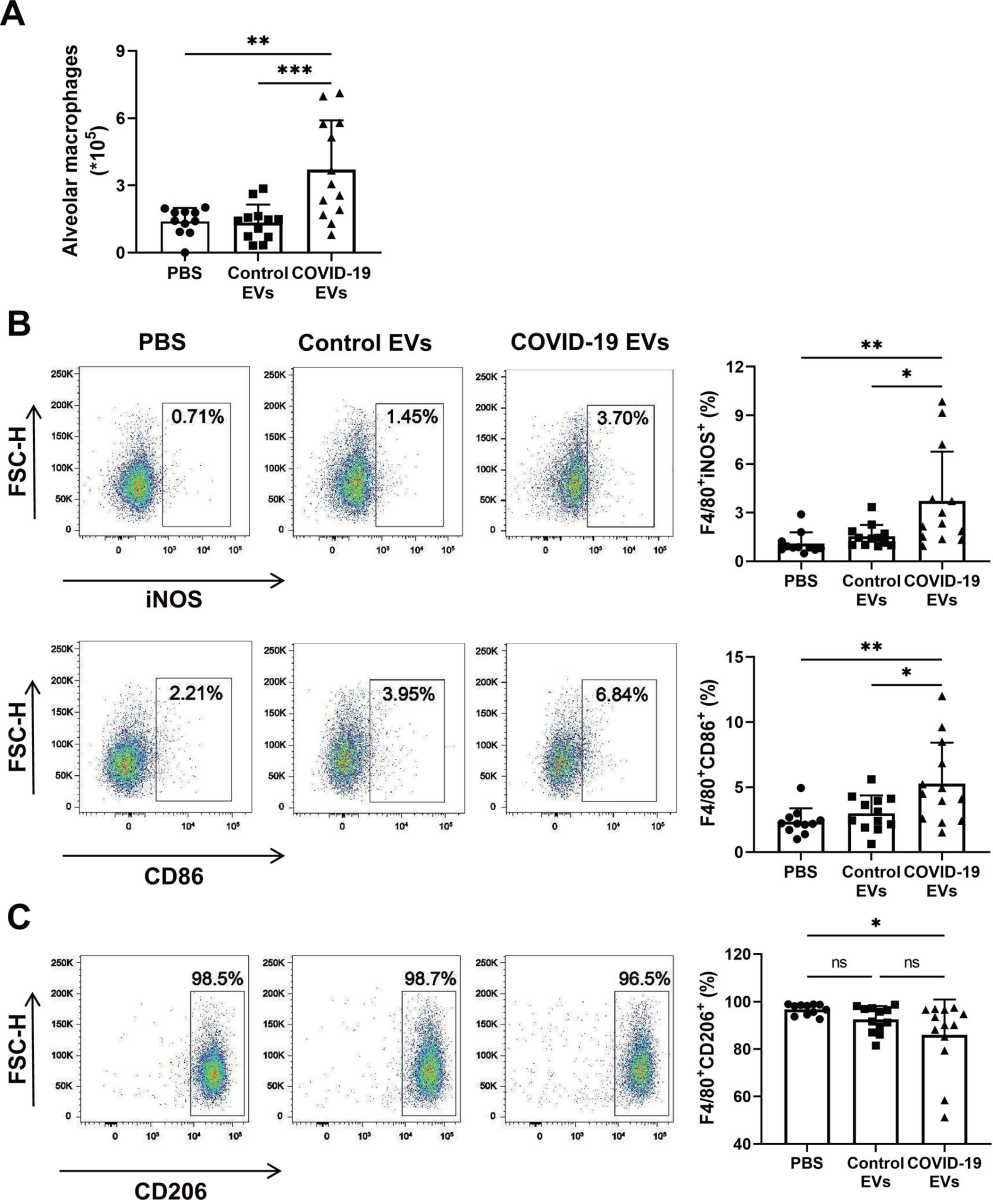
Circulating EVs from severe COVID-19 patients prompted M1 macrophage polarization *in vivo*. C57BL/6 mice received intratracheal injection of PBS (50 µL), control EVs (100 µg/50 µL), or COVID-19 EVs (100 µg/50 µL). BAL was collected at 24 h and centrifuged to collect cell pellet. BAL cells were stained with a combination of fluorescently labeled antibodies (BV785 anti-CD206, PE-cy7 anti-iNOS, PE anti-F4/80, and BV650 anti-CD86 or the isotype control) for 30 min. (**A**) Total alveolar macrophages in BAL were identified as F4/80+ in flow cytometry. (**B**) Representative flow cytometry plots display iNOS+ and CD86+ cells (M1) in gated F4/80+ macrophages. (**C**) Representative flow cytometry plots display CD206+ cells (M2) in gated F4/80+ macrophages. The percentage of cells in a plot represents the result of that particular experiment. Macrophage polarization data were summarized in the right panels. Data are expressed as mean ± SD, *n* = 11–13. *P < 0.05 and **P < 0.01. ns, not significant.

The M1 polarization of macrophages is critical in the pathogenesis of acute lung inflammation by secretion of pro-inflammatory cytokines and recruitment of neutrophils (23). At 24 h post-EV administration, BAL samples from the above experiment were subjected to phenotypical analysis with flow cytometry. COVID-19 EVs increased the quantity of alveolar macrophages (F4/80+) compared to control EVs and PBS groups (Fig. 3A) (gating strategy in Fig. S2). There was a significant elevation in the expression of F4/80+iNOS+ and F4/80+CD86+ cells in response to COVID-19 EVs, indicating M1 macrophage polarization (Fig. 3B). Compared with control EVs, treatment of COVID-19 EVs caused a trend toward reduction in the expression of F4/80+CD206+, a marker for M2 macrophage polarization; however, the reduction did not reach statistical significance (Fig. 3C). In addition, there was no difference between control EVs group and PBS group in all assays.

### Alteration of phenotypes of BMDMs by COVID-19 EVs *in vitro*

**Fig 4:**
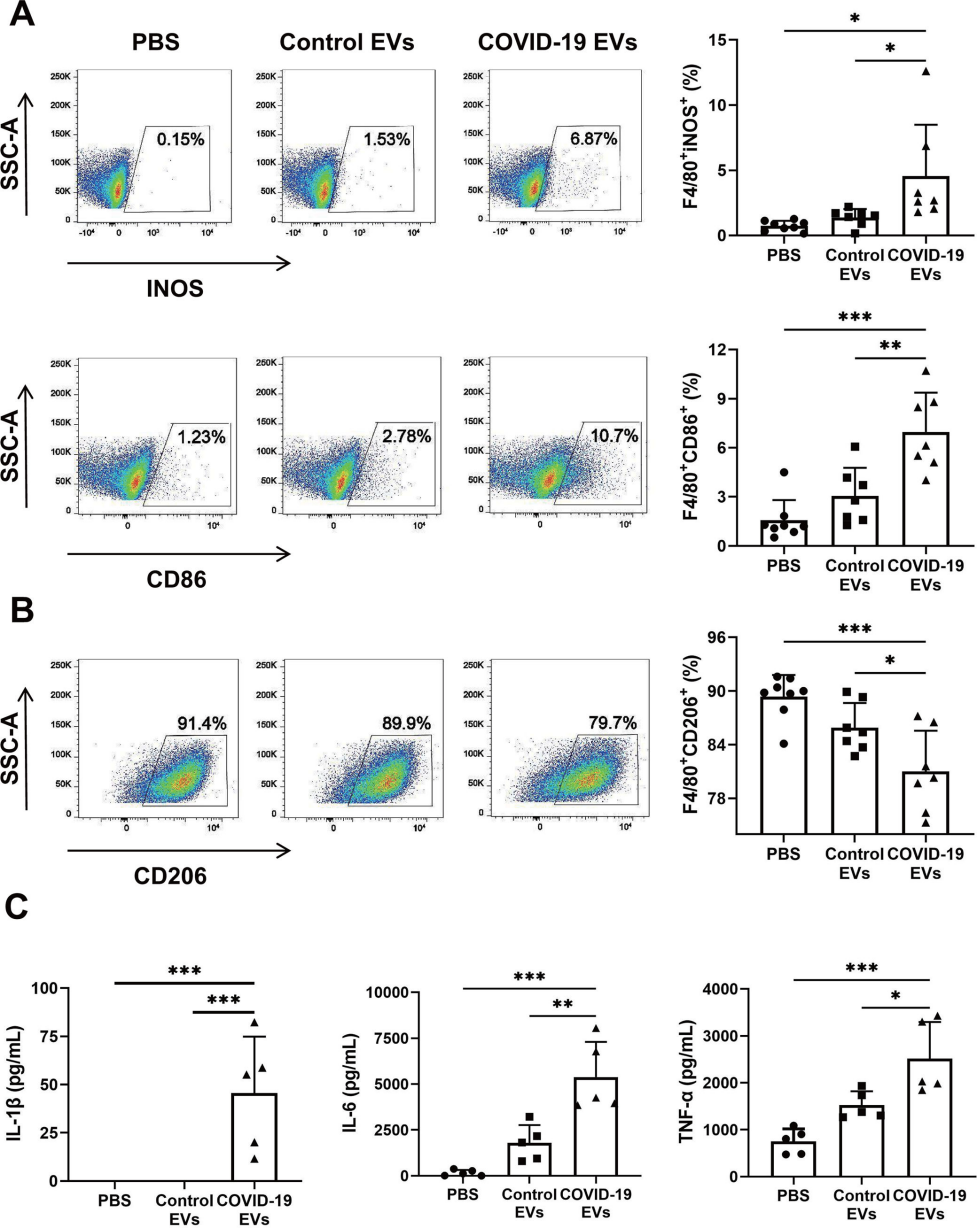
Circulating EVs from severe COVID-19 patients triggered M1 macrophage polarization *in vitro*. Mouse BMDMs were subjected to treatment with PBS, control EVs (100 µg/mL), or COVID-19 EVs (100 µg/mL). After 24 h, cells were stained with a combination of fluorescently labeled antibodies (BV785 anti-CD206, PE-cy7 anti-iNOS, PE anti-F4/80, and BV650 anti-CD86 or the isotype control) for 30 min. (**A**) Representative flow cytometry plots displayed iNOS+ and CD86+ cells (M1) in gated F4/80+ macrophages. *n* = 7–8. (**B**) Representative flow cytometry plots displayed CD206+ cells (M2) in gated F4/80+ macrophages. *n* = 7–8. The percentage of cells in a plot represents the result of that particular experiment. Macrophage polarization data were summarized in the right panels (**A and B**). (**C**) At 24 h, the culture supernatant was harvested and subjected to examination via ELISA for levels of IL-1β, IL-6, and TNF-α. *n* = 5. Data are expressed as mean ± SD. *P < 0.05, **P < 0.01, and *** *P* < 0.001.

To examine whether COVID-19 EVs elicit M1 macrophage polarization *in vitro*, mouse BMDMs were treated with PBS, control EVs, or COVID-19 EVs (100 µg/mL). After 24 h, flow cytometry was employed to analyze macrophage phenotypes. The expression of F4/80+iNOs+ and F4/80+CD86+ M1 macrophages was significantly higher in response to COVID-19 EVs (Fig. 4A) (gating strategy in Fig. S3). Conversely, COVID-19 EVs significantly reduced the quantity of F4/80+CD206+ M2 macrophages (Fig. 4B). To determine the effects of COVID-19 EVs on inflammatory response *in vitro*, the secreted levels of proinflammatory mediators in the culture medium were examined by ELISA. Results showed that levels of IL-1β, IL-6, and TNF-α were significantly elevated after treatment with COVID-19 EVs (Fig. 4C).

## DISCUSSION

To our knowledge, this study is the first to reveal that plasma EVs from severe COVID-19 patients elicit lung inflammation in an animal model. This assertion is substantiated by the following observations: (i) intratracheal delivery of plasma EVs from severe COVID-19 patients, but not those from control subjects, triggered acute lung injury in mice (Fig. 2). (ii) COVID-19 EVs drove alveolar macrophages toward M1 phenotype *in vivo* (Fig. 3). (iii) Primary macrophages exposed to COVID-19 EVs exhibited characteristics of M1 polarization (Fig. 4).

While the pathogenesis mechanisms of COVID-19 EVs have not been fully defined, several studies have examined their effects *in vitro*. COVID-19 EVs utilized ICAM-1 to engage with T cells. EVs from mild but not severe COVID-19 patients inhibited T cell metabolism and effector function (19). EVs from severe COVID-19 patients enhanced caspase 3/7 activity and promoted apoptosis in pulmonary endothelial cells compared to those from the asymptomatic group (24). ACE2-enriched EVs prompted the infection of live SARS-CoV-2 into Vero cells (25). However, another group reported that ACE2-containing EVs inhibited the entry of SARS-CoV-2-S-pseudotyped lentivirus into 293FT cells (26). Sur et al. (27) showed that EVs from severe COVID-19 patients induced the expression of NLRP3 and caspase-1 in endothelial cells compared to those from mild patients and healthy controls. In addition, the induction of IL-1β secretion was observed in endothelial cells.

In the present study, our results revealed that COVID-19 EVs triggered the polarization of M1 alveolar macrophages and the production of proinflammatory cytokines. These findings are consistent with the existing literature. Proteomic analysis of COVID-19 EVs identified Von Willebrand factor and serum amyloid A-2 as among the most significant differentially expressed proteins across severity groups (28). Both Von Willebrand factor and serum amyloid A-2 have been previously reported as inducers of M1 macrophage polarization (29, 30). EVs from severe COVID-19 patients had higher levels of IL-6, a proinflammatory cytokine, compared with those from healthy controls (22). COVID-19 EVs were shown to carry double-stranded RNA of the virus and triggered the release of proinflammatory cytokines in human peripheral blood mononuclear cells (31). Compared with EVs from COVID-19 pneumonia, EVs from COVID-19 ARDS exhibited decreased expression of miR-3168 and let-7e-5p, both of which target IL-8, a key chemokine for neutrophil recruitment to the lungs (32).

Effects of circulating EVs on proinflammatory response and lung inflammation have also been reported in sepsis. Mastronardi et al. (33) showed that circulating EVs from septic shock patients induced the expression of proinflammatory proteins, such as NF-ĸB in the lungs and heart of mice. Xu et al. (34) reported that circulating EVs from septic mice induced a proinflammatory response in BMDMs via the TLR7-MYD88 pathway. Hazelton et al. (35) found that plasma EVs from mice challenged with LPS exacerbated acute traumatic brain injury. Li et al. (36) revealed that EVs from sepsis patients promoted inflammation and apoptosis of macrophages, lung epithelial cells, and lung endothelial cells via delivery of miR-210-3p. A meta-analysis revealed that a big percentage of severe COVID-19 patients fulfilled the Sepsis-3 definition with concomitant organ dysfunction (37). Another study showed that SARS-CoV-2-associated sepsis was prevalent and had a higher mortality rate compared to bacterial sepsis in the early phase of the pandemic (38).

The present study suggests that the cargoes within COVID-19 EVs promote pro-inflammatory responses in alveolar macrophages, resulting in lung injury. Our findings carry broader implications, as COVID-19 EVs are also believed to induce injury in other organs, such as the kidneys (39). We hypothesize that the S and N proteins of SARS-CoV-2 present in these EV cargoes may participate in the lung injury. High levels of S and N proteins were detected in COVID-19 EVs during the acute phase of infection (20). Studies from our group and others have demonstrated that S and N proteins are able to induce acute lung injury even in the absence of an intact virus (40, 41). Both S and N proteins activate the NLRP3 inflammasome (42, 43), which may explain an earlier finding that endothelial cells exposed to COVID-19 EVs exhibited activation of the NLRP3 inflammasome (27). Additionally, plasma N protein levels were associated with the development of severe COVID-19 in hospitalized patients (44). Thus, pharmacological approaches, such as engineering cargoes of the exogenous and endogenous EVs, may have the potential to alleviate acute lung injury in COVID-19.

The present study has several limitations. First, this study included only 13 severe COVID-19 patients. It is essential to have a large sample size to corroborate the current findings. Second, we did not investigate the molecular composition of COVID-19 EVs. Analysis of the contents of EVs showed abundance of inflammatory, procoagulatory, and immunoregulatory proteins, differentiating COVID-19 patients from healthy controls as well as moderate severity from the critically ill. EN‐RAGE (S100A12), TF, and IL‐18 receptor 1 exhibited a strong association with disease severity and duration of hospital stay (24). Guervilly et al. (45) documented that patients with severe COVID-19 had increased TF activity in EVs and higher procoagulant activity compared to septic shock patients. Third, we did not explore the origins of circulating EVs that caused lung inflammation. Cappellano et al. (46) reported that platelet-derived EVs were higher in COVID-19 patients than non-COVID-19 patients and healthy controls, making them a distinctive feature of COVID-19 infection. Yim et al. (47) showed that Spike S1^+^ EVs likely originated from endothelial cells infected with SARS-CoV-2 and were correlated with disease progression and immune response.

In conclusion, our preliminary findings suggest a direct role of circulating EVs in lung injury among patients with severe COVID-19. Therapeutic approaches targeting EVs may represent a promising avenue in the fight against COVID-19.

## SUPPLEMENTAL MATERIAL

Supplemental figures msphere.00764-24-s0001.pdf Figures S1-S3.

ASM does not own the copyrights to Supplemental Material that may be linked to, or accessed through, an article. The authors have granted ASM a non-exclusive, world-wide license to publish the Supplemental Material files. Please contact the corresponding author directly for reuse.
