# Supplementary material for: Circulating extracellular vesicles from severe COVID-19 patients induce lung inflammation
Source: mSphere. 2024 Oct 30;9(11):e00764-24. doi: 10.1128/msphere.00764-24 (PMC11580465; doi:10.1128/msphere.00764-24)
Supplement: Supplemental figures — Figures S1-S3. [file msphere.00764-24-s0001.pdf]

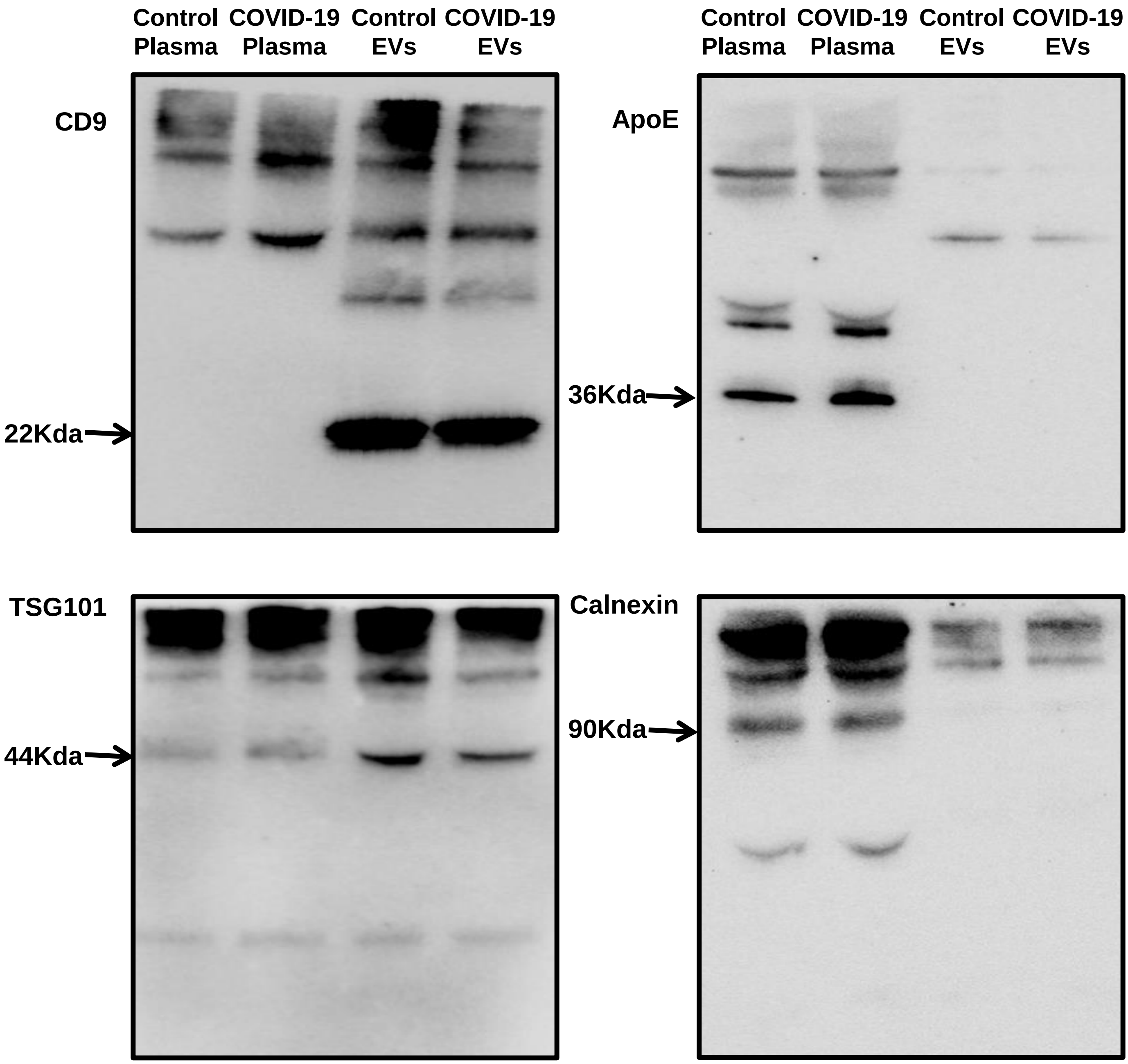

Figure S1. Evaluation of EV markers (CD9 and TSG101) and non-EV markers (ApoE and calnexin) in total plasma and EVs by Western blot.

Figure S1

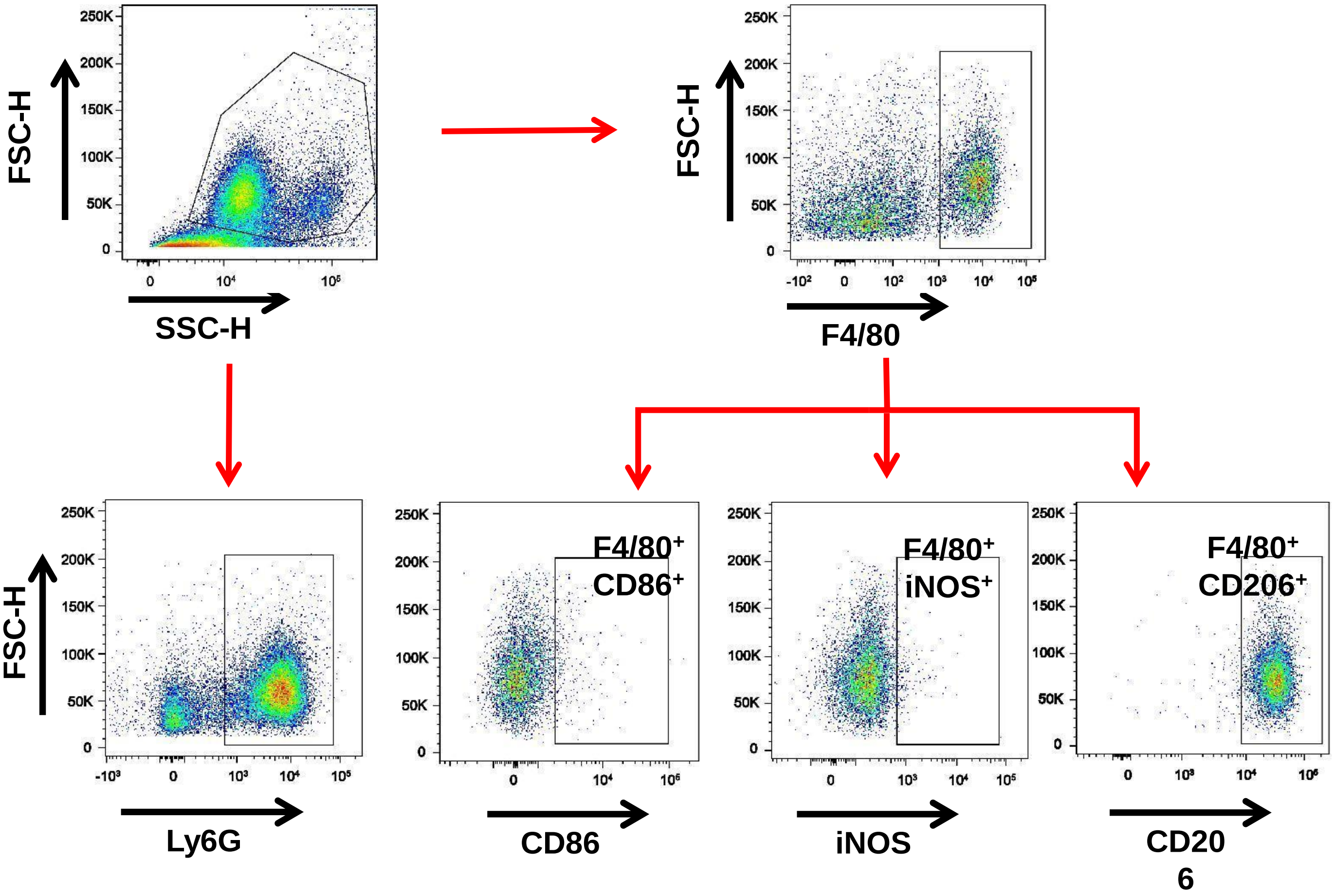

Figure S2. Gating strategy for determining neutrophils (Ly6G+) as well as M1 (F4/80+iNOS+ and F4/80+CD86+) and M2 alveolar macrophages (F4/80+CD206+) in the BAL.

Figure S2

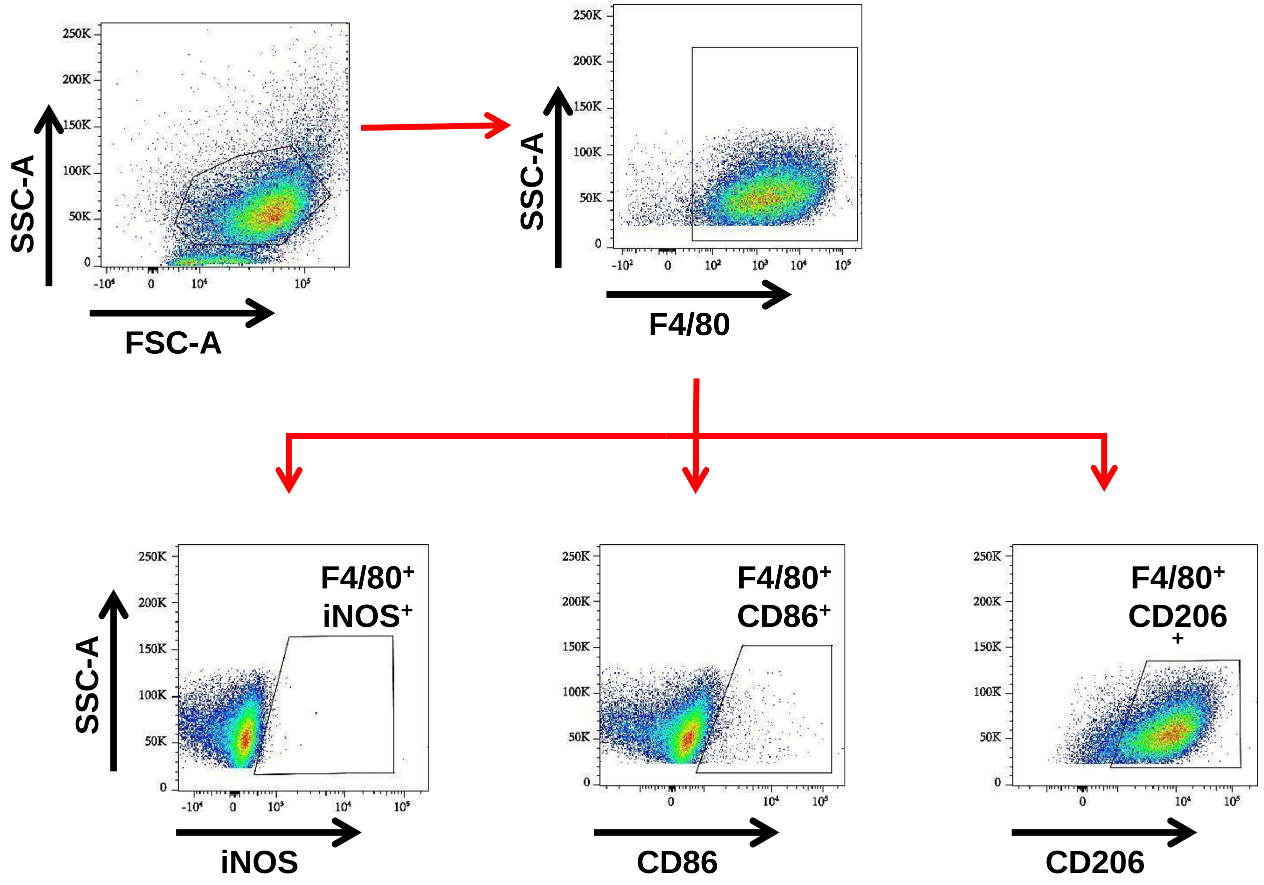

Figure S3. Gating strategy for determining M1 (F4/80+iNOS+ and F4/80+CD86+) and M2 macrophages (F4/80+CD206+) in BMDMs.

Figure S3
